# Seabird acoustic communication at sea: a new perspective using bio-logging devices

**DOI:** 10.1038/srep30972

**Published:** 2016-08-05

**Authors:** Andréa Thiebault, Pierre Pistorius, Ralf Mullers, Yann Tremblay

**Affiliations:** 1 Department of Zoology, Nelson Mandela Metropolitan University, South Campus, PO Box 77000, Port Elizabeth 6031, South Africa; 2 Marine Apex Predator Research Unit, Institute for Coastal and Marine Research, Nelson Mandela Metropolitan University, South Campus, PO Box 77000, Port Elizabeth 6031, South Africa; 3 DST/NRF Centre of Excellence at the Percy FitzPatrick Institute, Department of Zoology, Nelson Mandela Metropolitan University, South Campus, PO Box 77000, Port Elizabeth 6031, South Africa; 4 Department of Biodiversity, University of Limpopo, Private Bag X1106, Sovenga 0787, South Africa; 5 Institut de Recherche pour le Développement, UMR MARBEC 248: Marine Biodiversity, Exploitation and Conservation, Avenue Jean Monnet CS 30171, 34203 Sète cedex, France

## Abstract

Most seabirds are very noisy at their breeding colonies, when aggregated in high densities. Calls are used for individual recognition and also emitted during agonistic interactions. When at sea, many seabirds aggregate over patchily distributed resources and may benefit from foraging in groups. Because these aggregations are so common, it raises the question of whether seabirds use acoustic communication when foraging at sea? We deployed video-cameras with built in microphones on 36 Cape gannets (*Morus capensis*) during the breeding season of 2010–2011 at Bird Island (Algoa Bay, South Africa) to study their foraging behaviour and vocal activity at sea. Group formation was derived from the camera footage. During ~42 h, calls were recorded on 72 occasions from 16 birds. Vocalization exclusively took place in the presence of conspecifics, and mostly in feeding aggregations (81% of the vocalizations). From the observation of the behaviours of birds associated with the emission of calls, we suggest that the calls were emitted to avoid collisions between birds. Our observations show that at least some seabirds use acoustic communication when foraging at sea. These findings open up new perspectives for research on seabirds foraging ecology and their interactions at sea.

Sounds are widely produced within the animal kingdom and through technological advances these have increasingly been used to address questions of ecological importance. For example, the mechanisms of sound production and detection in insects can now be studied through the use of cutting-edge imaging techniques^1^,^2^. At a much larger scale, autonomous acoustic recorders coupled with adapted signal analyses provide powerful tools to assess and monitor ecosystem biodiversity^3^,^4^,^5^. In the field of animal behaviour, miniature microphones mounted on free-ranging small mammals have been used to record their vocalizations in their natural environment^6^. Studies on acoustic communication in seabirds have, however, only been conducted at their breeding colonies, where they are accessible on land (e.g.^7^,^8^).

Seabirds forage at sea but breed on land, where most species gather in colonies^9^. When commuting to and from the colony, they use calls for kin (parent-offspring) and partner recognition. These functions of acoustic communication have been demonstrated for various seabird families, including Spheniscidae^7^,^10^, Laridae^11^,^12^, Stercorariidae^13^, Procellariidae^8^ and Sulidae^14^.

At sea, species from all Orders of seabirds have been observed foraging in groups over patches of prey, most often in multi-species associations (e.g.^15^,^16^,^17^,^18^). Although group foraging could entail competition for the same resources, seabirds may also benefit from group associations^19^. Successive attacks from different predators disturb the prey aggregation^20^ and can result in an increase in individual foraging success^21^. Synchronous behaviours have been described in various species^16^,^17^,^18^,^22^,^23^, suggesting this may constitute a common strategy among marine predators to forage on schooling prey^21^. Social interactions are therefore central in the foraging ecology of a number of seabird species. Individuals may interact using various sensory channels, including visual or auditory. The marine environment is wide and open so vision is not obstructed and the information of the presence of individuals is transferred passively. However, the vast expanse poses challenges for acoustic communication. Sound levels in air decrease rapidly with distance and the presence of wind and waves may alter its transmission^24^,^25^. Whether seabirds actively signal their presence or exchange information through vocal communication when foraging at sea remains unknown.

We here investigated the use of acoustic communication by seabirds when at sea. We used Cape gannets as a model species as they have been shown to strongly rely on social interactions in their foraging strategies^21^,^26^,^27^. Based on observations from bird-borne video-cameras with built-in microphones, we described the context and behaviour of the birds when calling along their foraging trip.

## Results

**Figure 1:**
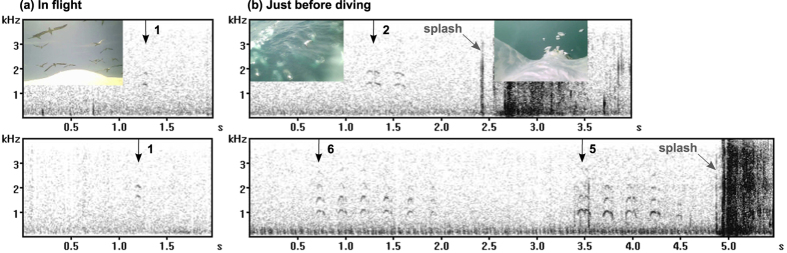
Examples of calls emitted by four different Cape gannets in flight (**a**) and diving (**b**) contexts. Calls and number in the sequence are indicated with black arrows, diving splash sound in grey.

We successfully collected video and GPS data from 35 foraging Cape gannets, and at least one call was recorded for 34 of them. During 42 h 15 min of video recording of Cape gannet behaviour at sea (excluding records at the colony), calls were recorded from 16 birds on 72 occasions. Calls were emitted in sequences composed of short calls (about 0.05 to 0.3 s), repeated one to 18 times every ~0.3 s, sometimes with longer gaps of ~1.5 s in between (Fig. 1). Most of the energy was concentrated between 300 and 2400 Hz, but the detailed structure of harmonic series could not be observed due to the poor quality of the camera microphones and the waterproof casing.

**Figure 2:**
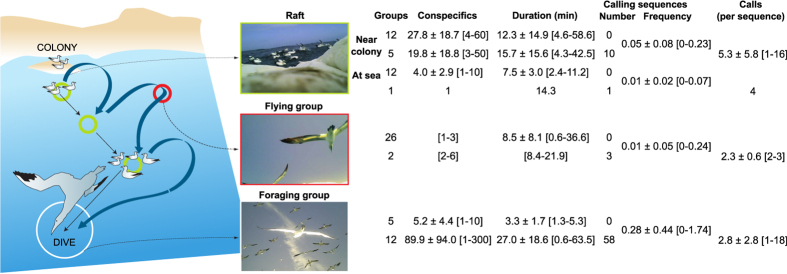
Context of calls emitted by Cape gannets along their foraging trips, from the colony to the first patch of food. Events (in the context of rafts, flying or foraging groups) were included only if the equipped bird was observed associated with conspecifics. For each context, the first and second rows in the table inform about events with no call and with calls, respectively. Numbers of conspecifics, duration of events, frequency of calling sequences (per minute) and number of calls within a sequence are shown as: mean ± standard deviation [minimum − maximum]. Figure adapted from^26^, with drawing designed by Pierre Lopez.

Figure 2 illustrates the context of call emissions along the foraging trips of Cape gannets. Calls were emitted only in the presence of conspecifics and mainly when the birds were foraging (81% of the total number of calling sequences). The frequencies of calling sequences (number per minute) were significantly different among the four contexts, i.e. when the birds were sitting in a raft nearby the colony, sitting in a raft at sea, flying in a group or foraging in a group (Kruskal-Wallis Rank Sum test, n1 = 17, n2 = 11, n3 = 16, n4 = 14, p < 0.001). However, no significance was found if including only the contexts of sitting in a raft nearby the colony or at sea, and flying in a group (Kruskal-Wallis Rank Sum test, n1 = 17, n2 = 11, n3 = 16, p = 0.27), showing that it is the distribution of frequencies in a foraging context that triggered the result obtained in the previous test. The frequency of calling sequences was on average higher when the birds were foraging (0.28) than when in any other context (between 0.01 and 0.05).

**Figure 3:**
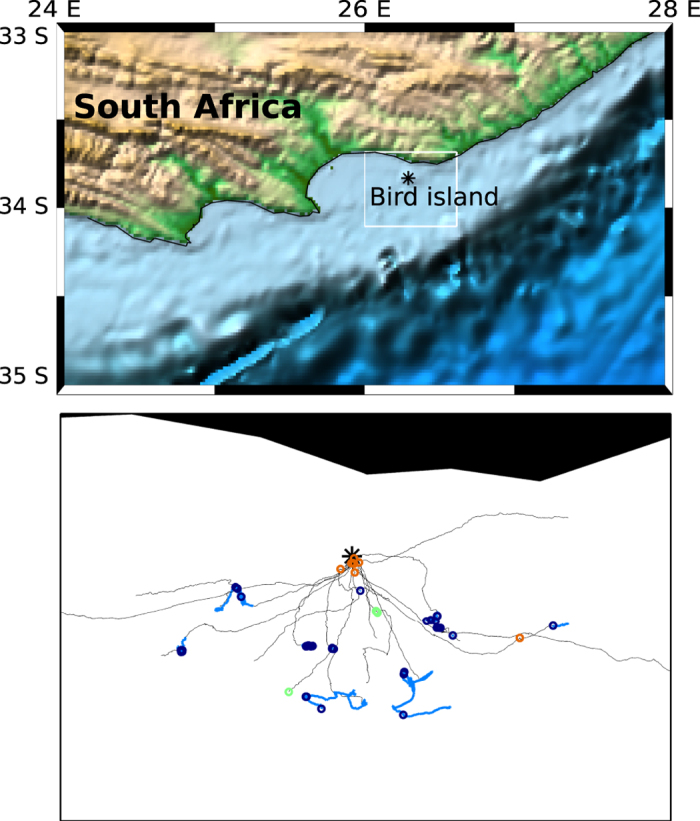
Location of calls along the trajectories of foraging Cape gannets. Top panel: map of the study area. The background was obtained from the “Etopo 1 Ice Full” image file available at https://www.ngdc.noaa.gov/mgg/. The black star indicates the location of the colony where the birds were equipped (Bird Island in Algoa bay, South Africa). Bottom panel: portion of tracks for which video records were available. Foraging events along the trajectories are represented in light blue (thick grey). The black start indicates the location of the colony. Circles indicate the location of calling sequences emitted in a group of flight (green or light grey), in a raft (orange or medium grey), or during a foraging event (blue or dark grey). For clarity reason, the calls recorded from two birds (all during a foraging event) are not represented on the map because they were emitted much further away. The events and calling sequences were located to the closest point in time on the movement data. The figure was generated with the MATLAB software and the Mapping toolbox^32^, using the Mercator projection.

Figure 3 shows the geographical location of calls emitted along the foraging trips of Cape gannets. Calls in rafts were mostly located within a few kilometres around the colony. The equipped birds all foraged at different locations so that no spatial pattern was observed as to where the calls were emitted within the foraging area.

### Near-colony raft

After leaving the colony, the study birds first landed on the water to preen, thereby forming near-colony rafts^26^. Ten calling sequences were recorded during five out of 17 of these near-colony rafts, at an average frequency of 0.05 per minute. One single call was emitted just before a bird landed on the water. Four calling sequences were emitted when birds were sitting on the water: three single calls and one sequence made of eight calls. Single calls were associated with a jerky camera movement. For one of those the footage showed another bird brushing past (Supplementary Video S1). In addition, five birds emitted calls when taking off to leave the raft on their foraging trip, including a non-equipped bird that took off in the field of view of a deployed camera (Supplementary Video S2). Three of these sequences were made up of 3–4 calls, while the other two consisted of 15 and 16 calls (thus comprising two of the longest calling sequences recorded).

### Commuting

When flying in groups, the gannets were mostly quiet. Only three calling sequences (2–3 calls) were recorded from two out of 34 different flying groups (20 equipped individuals). Calls were emitted at an average frequency of 0.01 sequence per minute. Another gannet emitted a sequence of calls (composed of four calls) just before landing in a raft at sea.

### Foraging

In total, instrumented birds were observed within 17 different foraging aggregations. No calls were recorded for five of these foraging groups, which all consisted out of less than 10 individuals in size. Fifty eight calling sequences were recorded from individuals within the remaining 12 foraging groups. These included two small aggregations (less than 10 gannets) and all the nine large aggregations observed (of up to 300 gannets), in addition to a group foraging behind a fishing boat. The highest number of calls during foraging events also resulted in highest frequencies, with an average of 0.28 calling sequence emitted per minute.

Twenty eight calling sequences were recorded from seven birds that were flying around in the foraging flock. These were made up of one to three calls (Fig. 1a) and three of them were concomitant with a jerky camera movement. A number of the calls were emitted when the equipped bird initiated a dive, a few seconds before it hit the water (Fig. 1b; Supplementary Video S3). Twenty four dives performed by eight different birds (out of 100 dives recorded from 16 birds) were preceded with a calling sequence, made of two to 11 calls (3.7 of average). The remainder of the calls were emitted just before a gannet landed on the water (one sequence made up of two calls), when they were sitting on the water (three consisting of one to three calls) or when taking off after a dive (two consisting of two and 18 calls).

## Discussion

Our observations show that Cape gannets actively signal their presence when foraging at sea. They emitted calls exclusively when conspecifics were visible in their surroundings and they tended to call more when gathered in large groups. The calls were context-specific and associated with specific behaviours, showing that they perform specific functions.

Most of the energy of the recorded calls was under 2500 Hz, which is similar to vocalizations from King penguins *Aptenodytes patagonicus*^7^ or Black-headed gulls *Larus ridibundus*^11^, but lower than vocalizations from Blue-footed boobies *Sula nebouxii*^14^ when recorded at their colony.

The contexts in which calls were emitted by Cape gannets suggest strongly that some calls could be used as a means to avoid collisions or to respond to collisions. When the gannets were sitting in rafts or flying in flocks, we recorded very short calls simultaneous with jerky camera movements (Supplementary Video S1). These observations suggest that the calls were emitted as a response to collisions or near collisions. Similarly at the breeding colony, Cape gannets emit a call when coming into contact with conspecifics in flight, a behaviour observed more frequently when they fly in dense flocks (pers. obs.). In addition, we recorded Cape gannets calling when taking off from a raft (Supplementary Video S2), a signal possibly used to warn other birds to stay out of the flyway path. These calling sequences were longer than the ones emitted when the birds were sitting or flying, with calls repeated at least twice (up to 18 times). Repeating a warning signal can be useful as it increases the probability for the information to be received. The calls emitted just before diving could also function as a warning for surrounding individuals. The gannets initiate a dive from up to 30 m height and they can hit the water at 24 m/s^28^. It has been shown that a collision with a plunge-diving gannet can be fatal for both birds^29^. Avoiding collision with plunging individuals may hence be crucial for the gannets to fully benefit from group foraging^21^. In this context, the number of calls repeated (from zero for dives not preceded with a call, up to 11) may depend on the number and density of birds present in the immediate proximity of the plunging gannet. The number of repeated calls may also depend on the weather, with more calls emitted in windy conditions in order to overcome background noise^30^.

We do not know at which distance these calls can be perceived by seabirds, but it appears unlikely to be greater than the distance at which they can perceive individuals visually. Calls might therefore be confined to short range communications (relative to their foraging range) and not be used to actively inform other seabirds of the individual’s presence or the presence of prey. However, when vision is impaired (at night or in foggy weather for example), calls might serve to inform other seabirds at sea. As calls tended to be more frequent or repeated with group size, larger aggregation must be noisier and therefore more detectable. At night, Cape gannets typically form rafts that last all night long. Because of currents and winds, these rafts must probably be maintained actively to avoid individual dispersion. Acoustic communication could be key in this context.

To our knowledge, this is the first study describing acoustic communication of seabirds at sea. It is also the first use of bird-borne devices to analyse sound in seabirds. We demonstrated the use of calls as “honking in traffic”, but the poor quality of our recorders only allowed us to recognize and locate gannet calls in time. The analysis of the detailed structure of these calls will require sound recordings of better quality, potentially allowing an in-depth understanding of seabird sound repertoires. Devices are now available to record sound data of high quality using microphones that are robust and miniature enough to be deployed on wild small animals^6^. However, a major difficulty for studying acoustic communication in seabirds is due to the windy and noisy environment in which they move and forage. A solution to this could be the use of contact microphones that considerably improve the signal-to-noise ratio^31^. The remaining challenge for using microphones on seabirds is maintaining high quality recordings despite a waterproof casing. Despite these challenges, sound recording of seabirds at sea will open new perspectives on several aspects of their foraging ecology. Their behaviour and time-activity-budgets can be monitored along foraging trips from the typical splash sounds emitted when taking off from the water, landing on the water and diving. Compared to data from Time-Depth Recorders (TDR), the dive profiles are not captured with microphones, but they can be an interesting complement as they provide additional information on the behaviour of the birds. Seabirds often gather in groups, on land to breed^9^ and at sea to forage (e.g.^15^,^17^,^21^). Social interactions necessarily shape the behaviour and foraging strategies of seabirds. Advances in technologies and the development of bio-logging provide us with new tools to observe and study seabirds at sea and enhance our understanding on the role of social interactions in their ecology and behaviour.

## Methods

### Data collection

We deployed video cameras with built-in microphones and GPS units on 36 breeding Cape gannets at Bird Island (33^°^50′ 26.6″S, 26^°^17′ 14.5″E, Algoa Bay, South Africa) during the 2010–2011 breeding season. Fieldwork was conducted under a permit from South African National Parks (SANParks). All experimental protocols were approved by both SANParks and the Nelson Mandela Metropolitan University Ethics Committee (reference: A10-SCI-ZOO-008), and were carried out in accordance with the approved guidelines. The birds were captured near their nest, when departing to sea after a changeover with their partners. Only one adult per nest was equipped for one foraging trip, while the partner stayed at the nest guarding the chick. The video cameras (Camsports Nano; Camsports, Estrablin, France) recorded at a resolution of 736 × 480 pixels at 25 frames per second with a 74^°^ lens angle, for a maximum of 90 min (due to limited battery capacity). Microphones in the video camera loggers recorded the sound at a sampling frequency of 8 kHz. The GPS devices (i-GotU GT-600, Mobile Action Technology, Taipei, Taiwan) recorded a geographic position every 5 s when the birds moved at a speed higher than 10 km^∗^h^−1^ and every 10 s or 30 s otherwise. A handheld GPS was placed in front of the camera lens so that the Greenwich Mean Time was recorded on the footage. The video and sound observations were hence accurately synchronized to movement data using the satellite derived time. The handling process lasted less than eight minutes and included weighing individuals using a spring balance [Pesola, Baar, Switzerland; precision 50 g]) and device attachment through the use of adhesive tape (Tesa, Hamburg, Germany). The loggers were attached to the lower back of the birds in such a way that potential drag due to modification of the birds’ body shape was minimized. The total mass attached to a bird was 70–75 g, corresponding to 2.3–3.0% of the bird’s body mass (2,400–3,100 g, n = 36). The nests were then monitored regularly, and the study birds were recaptured soon after their return to the colony and the devices were retrieved.

### Data analysis

To analyse the images, video footage were observed frame by frame and the events of interest were visually flagged using a purpose-built video reader and event recorder coded in MATLAB software^32^. Video data provided information on the behaviour of equipped birds, including taking off, sea landing, and diving, from which we defined flights, sitting on the water and foraging events (defined as in ref. 27). The surroundings of the study bird were also observed, as were interactions with conspecifics and other predators (other seabird species, dolphins, and boats).

To analyse the sound, videos with sound recordings were played using VLC media player (VideoLAN, France) while simultaneously running a software designed to record observed behaviours. All vocalization events were flagged and recorded together with the time since the start of the video. The spectrograms of identified calls (using a Hamming function on a 256 points window size, 31 Hz frequency resolution, with 75% overlap) were then displayed using Avisoft-SASLab Lite (Avisoft Bioacoustics, Germany). Spectrograms were only used to confirm the emission of a call from a gannet, and no detailed analyses of acoustic signals were conducted because of poor quality of a number of the recordings (the microphones were embedded in a waterproof casing resulting in sound degradation). Observations from deployed individuals were included if at least one call was recorded.

The image and sound observations were located to the closest point in time on the movement data. Combining image and sound observations together with location data, we described the context and behaviour of the birds when calling along their foraging trip. Recorded calls were assumed to be emitted by the equipped bird (sound emission closer to the microphone), except if another individual was observed calling into the camera.

Statistics were computed using R software^33^. Means are shown ±standard deviation. The Kruskal-Wallis Rank Sum test was used to compare the distribution of the frequency of calling sequences in different contexts. If more than one value of frequency was available for an individual in a given context, only one value was randomly selected in order to reduce the effect of non-independence in the data.

## Additional Information

**How to cite this article**: Thiebault, A. *et al.* Seabird acoustic communication at sea: a new perspective using bio-logging devices. *Sci. Rep.* **6**, 30972; doi: 10.1038/srep30972 (2016).

## Supplementary Material

Supplementary Information

Supplementary Video S1

Supplementary Video S2

Supplementary Video S3
