# Supplementary material for: Seabird acoustic communication at sea: a new perspective using bio-logging devices
Source: Sci Rep. 2016 Aug 5;6:30972. doi: 10.1038/srep30972 (PMC4974508; doi:10.1038/srep30972)
Supplement: Supplementary Information [file srep30972-s1.pdf]

Supplementary Information for:

Seabird acoustic communication at sea: a new perspective using bio-logging devices.

Andréa Thiebault (1,2*), Pierre Pistorius (3,2), Ralf Mullers (4) and Yann Tremblay (5)

1. Department of Zoology, Nelson Mandela Metropolitan University, South Campus, PO Box 77000, Port Elizabeth 6031, South Africa
2. Marine Apex Predator Research Unit, Institute for Coastal and Marine Research, Nelson Mandela Metropolitan University, South Campus, PO Box 77000, Port Elizabeth 6031, South Africa
3. DST/NRF Centre of Excellence at the Percy FitzPatrick Institute, Department of Zoology, Nelson Mandela Metropolitan University, South Campus, PO Box 77000, Port Elizabeth 6031, South Africa
4. Department of Biodiversity, University of Limpopo, Private Bag X1106, Sovenga 0787, South Africa
5. Institut de Recherche pour le Développement, UMR MARBEC 248: Marine Biodiversity, Exploitation and Conservation, Avenue Jean Monnet CS 30171, 34203 Sète cedex, France

- Corresponding author: andrea.thiebault@gmail.com

Supplementary videos

Video S1. The camera is filming backwards, from the lower back of a Cape gannet. A call is emitted at 4 sec in the video, simultaneously with a jerky camera movement and an impact sound, and immediately followed by another bird brushing past. The spectrogram (displayed with Avisoft-SASLab Lite software) on top shows the call at 4.3 sec.

Video S2. The camera is filming backwards, from the lower back of a Cape gannet. Another gannet sitting on the water just behind the equipped bird takes off and emits a call at 5 sec in the video. The spectrogram (displayed with Avisoft-SASLab Lite software) on top shows the call at 5.6 sec.

Video S3. The camera is filming backwards, from the lower back of a Cape gannet. The equipped bird emits a call while initiating a dive. The spectrogram (displayed with Avisoft-SASLab Lite software) on top shows the calling sequence composed of a repetition of 11 calls (6 then 5) from 2.7 sec, followed by a splash sound at 6.9 sec when the bird hits and enters the water.
